# Age and gender effects on striatal dopamine transporter density and cerebral perfusion in individuals with non-degenerative parkinsonism: a dual-phase ^18^F-FP-CIT PET study

**DOI:** 10.1186/s13550-024-01126-1

**Published:** 2024-07-17

**Authors:** Ji-Young Kim, Seo Young Kang, Byung Seok Moon, Bom Sahn Kim, Jee Hyang Jeong, Hai-Jeon Yoon

**Affiliations:** 1 Department of Nuclear Medicine, College of Medicine, Ewha Womans University, Seoul, Republic of Korea; 2 Department of Neurology, College of Medicine, Ewha Womans University, Seoul, Republic of Korea

**Keywords:** Parkinsonism, Dopamine transporter, Cerebral perfusion, Dual-phase PET, ^18^F-FP-CIT, PET

## Abstract

**Background:**

Dual-phase fluorine-18 labeled N-3-fluoropropyl-2β-carbomethoxy-3β-(4-iodophenyl) nortropane (^18^F-FP-CIT) positron emission tomography (PET) scans could be used to support disorders like Parkinson’s disease (PD). Dopamine transporter (DAT) binding and cerebral perfusion are associated with ageing and gender. We investigated the effects of age and gender on non-degenerative parkinsonism, using automated quantification in striatum: specific binding ratios (SBRs) for DAT binding in delayed phase PET (dCIT) and standardized-uptake-value ratios (SUVRs) for cerebral perfusion in early phase PET (eCIT). We also examined the correlations between SBR and SUVR.

**Methods:**

This retrospective study analyzed subjects with dual-phase ^18^F-FP-CIT PET scans. The eCIT images were acquired immediately post-injection, and dCIT images were taken 120 min later. With Brightonix software, automated quantification of SBRs for dCIT and SUVRs for eCIT were acquired from visually normal scans. The effects of aging and gender were assessed by regressing SBRs and SUVRs on age for both genders. The correlations between SUVRs and SBRs were evaluated.

**Results:**

We studied 79 subjects (34 males and 45 females). An age-related reduction in SBRs was observed in the dorsal striatum, ventral striatum, caudate nucleus, and putamen for both genders. SUVRs were found to negatively correlate with age in the dorsal striatum, ventral striatum, caudate nucleus, and putamen for males and in the dorsal striatum and caudate nucleus for females. Positive correlations between SBRs and SUVRs in the dorsal striatum, ventral striatum, caudate nucleus, and putamen for male and in the dorsal striatum, caudate nucleus, and putamen for females.

**Conclusions:**

Using quantified values from dual-phase ^18^F-FP-CIT PET with a single injection, we demonstrate a negative impact of age on SBRs (DAT binding) in the striatum for both genders and SUVRs (cerebral perfusion) in the dorsal striatum and caudate nucleus for both genders and in the ventral striatum and putamen for males. Additionally, we found positive associations between SBR and SUVR values in the dorsal striatum, caudate nucleus, and putamen for both genders and in the ventral striatum for males.

**Supplementary Information:**

The online version contains supplementary material available at 10.1186/s13550-024-01126-1.

## Introduction

Parkinsonism encompasses a group of movement disorders characterized by bradykinesia, tremor at rest, rigidity, and postural instability [1, 2]. The signs and symptoms of parkinsonism most commonly arise from degeneration of the dopaminergic system, including in association with idiopathic Parkinson’s disease (PD), multiple system atrophy, progressive supranuclear palsy, corticobasal degeneration, and dementia with Lewy bodies. Conversely, forms of parkinsonism, such as essential tremor, drug-induced parkinsonism, vascular parkinsonism, and those of a psychogenic nature, are not associated with degeneration of the dopaminergic system [3, 4].

Dopamine transporter (DAT) imaging is used for diagnosing parkinsonism [4–8]. Presynaptic striatal DAT binding is highly correlated with the availability of dopaminergic neurons [4, 5]. DAT imaging such as fluorine-18 labeled N-3-fluoropropyl-2β-carbomethoxy-3β-(4-iodophenyl) nortropane (^18^F-FP-CIT) positron emission tomography (PET), has demonstrated high sensitivity and in detection degeneration of the dopaminergic system and is approved for clinical use [4–7]. Brain perfusion imaging are reported to enhance the differential diagnosis among parkinsonian disorders [9–11]. Obtaining nuclear images that assess both DAT binding and cerebral perfusion is highly beneficial for the differential diagnosis in individuals with parkinsonism [5, 10–12].

Using dual-phase ^18^F-FP-CIT scans with a single tracer enables the acquisition of images that effectively simulate the use of two tracers, providing additional information on both DAT binding and cerebral perfusion [11–13]. In dual-phase ^18^F-FP-CIT scans, the delayed phase ^18^F FP-CIT (dCIT) can detect degenerative parkinsonism by interpreting striatal DAT binding, while several studies have reported that eCIT could be used for supporting the differential diagnosis of parkinsonism [5, 10–12]. Recent studies suggest that dual-phase ^18^F-FP-CIT PET imaging comprising dCIT and eCIT scans, should be performed to increase diagnostic accuracy in clinical practice. The dual-phase ^18^F-FP-CIT scan is an invaluable tool for obtaining striatal DAT binding in dCIT and observing cerebral perfusion in eCIT [12, 13].

It is well known that human aging is associated with reductions in brain activity and the integrity of gray and white matter, which are assessed by functional imaging, modalities such as PET and magnetic resonance imaging (MRI) [14, 15]. DAT binding and cerebral perfusion are closely associated with age. DAT binding has consistently demonstrated ageing effects with multiple tracers in healthy controls using PET [16–22]. Furthermore, gender differences in striatal DAT activity in PD have been observed, with females exhibiting higher age-related DAT binding and PD occurring more frequently in males than in females [23–26]. In several studies, global cerebral perfusion has significantly declined with age in healthy volunteers [20–22]. In regional cerebral perfusion, gender-related differences have also been noted in the normal controls [27–30]. Age and gender are important factors to consider when observing striatal activity in parkinsonism.

This study aimed to investigate the effects of age and gender on striatal DAT binding and cerebral perfusion in individuals with non-degenerative parkinsonism using dual-phase ^18^F-FP-CIT images in striatal subregions. We used the quantified values as a fast and easy-to-use method for quantification of DAT binding and cerebral perfusion. We demonstrated the changes in specific binding ratios (SBRs) for DAT binding and in standardized-uptake-value ratios (SUVRs) for cerebral perfusion with respect to age and gender in striatal subregions using quantified data. We also assessed the correlation between SBRs for DAT binding and SUVRs for cerebral perfusion in striatal subregions for male and female, respectively. This study can provide insights into changes in striatal DAT binding and cerebral perfusion according to age and gender using dual-phase ^18^F-FP-CIT scans with only a single tracer.

## Materials and methods

### Subjects

This retrospective study included subjects who underwent ^18^F-FP-CIT PET scans between April 2018 and September 2022 at Ewha Womans University Mokdong Hospital and between March 2019 and December 2021 at Ewha Womans University Seoul Hospital. Dual-phase ^18^F-FP-CIT PET images and clinical data including age, sex, onset date, and final diagnosis were obtained. Only subjects with normal findings on dCIT and eCIT were included. All subjects underwent MRI scans, with the scanning interval between PET and MRI being less than 1 year. Subjects with a history of stroke, lacunar infarction, brain tumor, brain metastasis, history of brain surgery, and other brain occupying lesions observed in their MRI scans were excluded. The subjects were followed for more than 2 years after parkinsonism onset, and the final diagnosis was non-degenerative parkinsonism. The subjects were discontinued medications which could significantly influence the dopamine transporter binding ligands prior to imaging at least five half-lives according to the protocol [14, 15]. The institutional review board of Ewha Womans University Hospital (No. 2024-02-020) approved the study and waived the requirement for informed consent due to the retrospective study design.

### Dual-phase ^18^F-FP-CIT imaging acquisition protocol

A single dose of 185MBq ± 18.5 was administered to all subjects as an intravenous injection. The eCIT images were acquired immediately after injection for 10 min and the dCIT images were acquired 120 min after injection for 10 min using dedicated PET/computed tomography (CT) scanners (Biograph mCT128 Siemens Healthcare, Germany and Discovery MI, GE Healthcare, USA) [7, 16–18]. 21 subjects used the Biograph mCT128 (Siemens Healthcare), while 59 subjects used the Discovery MI (GE Healthcare). CT scans were performed for attenuation correction, followed by an emission PET scan of the brain. The CT parameters were as follows: voltage of 100kVp, current of 35mAs, slice thickness of 1.0 mm, rotation time of 1.0s, and pitch of 1.0 s PET images were reconstructed using TrueX and Gaussian filtration for eCIT and iterative algorithms and an all-pass filter for dCIT with a 512 × 512 matrix.

### Visual interpretation of ^18^F-FP-CIT PET

All ^18^F-FP-CIT images were visually assessed by two expert readers, who were experienced nuclear medicine physicians with 7 and 14 years of experience, respectively. The readers performed visual interpretations of axial, coronal, and maximum-intensity-projections ^18^F-FP-CIT PET images according to practice guidelines and were blinded to clinical information. The visual interpretation criteria for dCIT were based on DAT binding in the striatum classified as either decreased or preserved DAT binding. Preserved DAT binding images show a symmetric and homogeneous pattern without a deficit in the bilateral striatal system. We considered the eCIT images to be normal when they exhibited symmetric and homogeneous radioactivity without deficits in the brain [5, 19]. Visual interpretations with discrepancies between dCIT and eCIT were all excluded from this study.

### Quantitative analyses

Using Brightonix sofrware (https://brtnx.com/en/, South Korea), we acquired automated quantified values of dual-phase ^18^F-FP-CIT images, including both SBRs of dCIT and SUVRs of eCIT. Brightonix software is artificial intelligence (AI)-based and provides automated quantified values by directly processing reconstructed DICOM (digital imaging and communications in medicine) ^18^F-FP-CIT PET images without the need for any anatomical images [20]. All reconstructed PET images were spatially normalized to a Montreal Neurological Institute (MNI) standard space using an in-house software template. Automatic quantitative analyses were based on volumes of interest, which were defined on atlas templates from the Melbourne Striatal for dCIT and from the Automated Anatomical Labeling Atlas 3 (AAL3) for eCIT, respectively (Supplementary Fig. 1).

In the acquired quantified values, we selected the striatal subregions such as the dorsal striatum (including the caudate nucleus and putamen) and ventral striatum for SBRs from dCIT and for SUVRs from eCIT. SBRs from dCIT were obtained using the occipital cortex as a reference region and SUVRs from eCIT were obtained using the cerebral white matter as a reference region to exclude the risk of abnormal cortical perfusion.

### Statistical analysis

Statistical analysis was conducted using SPSS Statics for Windows, version 27.0 (IBM Corp., Armonk, NY, USA). An independent two sample t-test was performed to assess differences in age, SBRs, and SUVRs differences between males and females. A normality test was conducted. Multiple regression was used to identify the relationships between age and SUVRs as well as between age and SBRs in striatal subregions for males and females that corrected for confounding effects of two different scanners, respectively. The correlations between SUVRs and SBRs were evaluated using Pearson correlation analyses in striatal subregions. The statistical threshold of the post-hoc analyses were Bonferroni corrected: *P* < 0.05/4 considering 4 comparisons (4 target regions: dorsal striatum, ventral striatum, caudate nucleus, and putamen). The values were expressed as mean ± standard deviation (SD). A *P*-value < 0.05/4 was considered statistically significant.

## Results

### Subjects

**Fig. 1:**
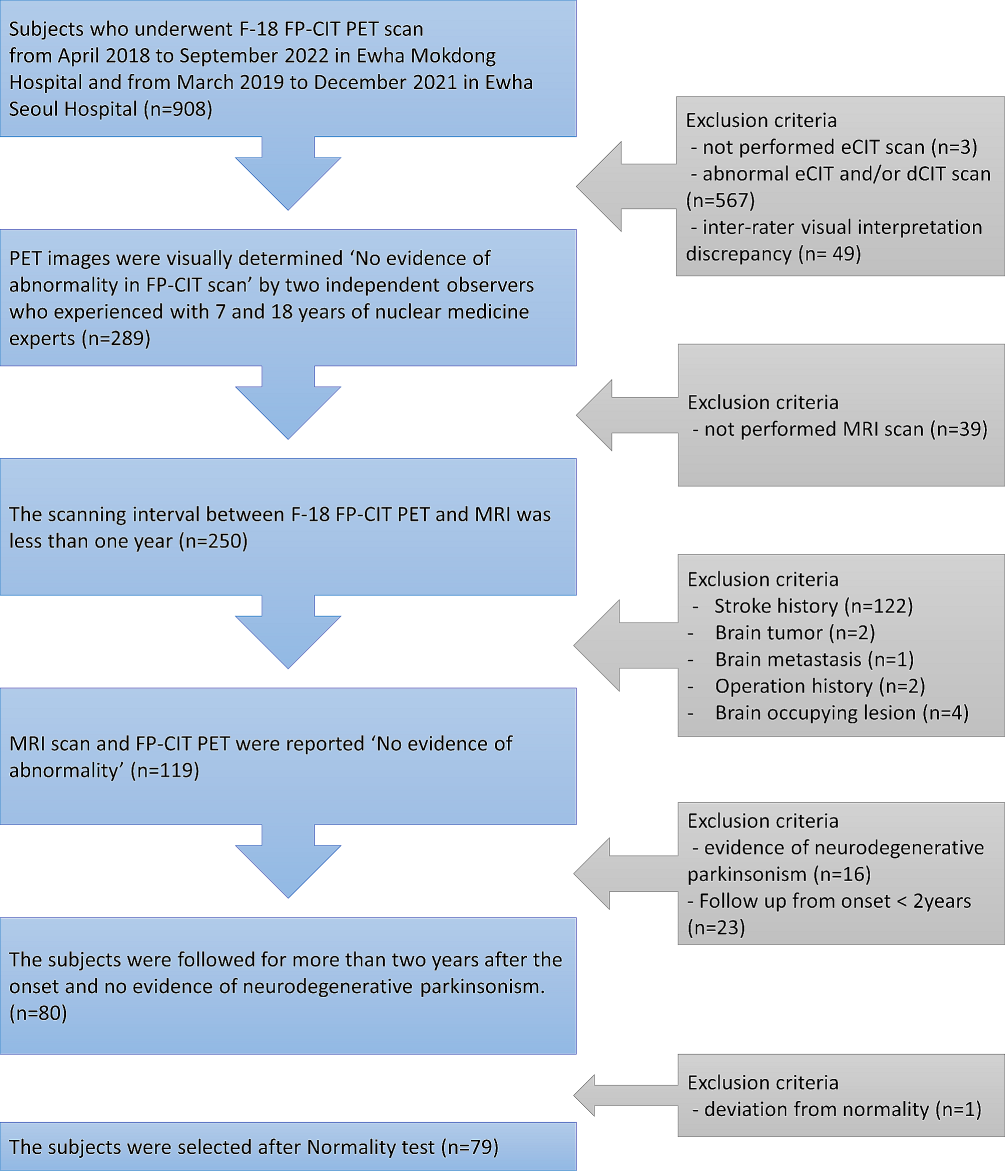
Patient selection schema

We collected data from 908 patients who underwent ^18^F-FP-CIT PET scans between April 2018 and September 2022 at Ewha Womans University Mokdong Hospital and between March 2019 and December 2021 at Ewha Womans University Seoul Hospital. The exclusion criteria for the 908 subjects were as follows: subjects who did not undergo eCIT scanning (*n* = 3), dual-phase ^18^F-FP-CIT images that were visually interpreted as having an abnormal dCIT and/or eCIT scans (*n* = 567), inter-rater disagreement for visual interpretation (*n* = 49), subjects without an MRI scan within a year before or after the PET scan (*n* = 39), abnormal findings in the MRI (*n* = 131), evidence of neurodegenerative parkinsonism (*n* = 16), follow-up from onset of less than 2 years (*n* = 23), and a deviation from normality (*n* = 1). Finally, a total of 79 subjects (34 males, age range 38–87 years, mean age ± SD: 70.4 ± 13.2 years; 45 females, age range 43–84 years, mean age ± SD: 69.4 ± 9.8 years) were selected for this study (Fig. 1). There was no significant age difference between males and females (*p* = 0.075). The final diagnoses of the subjects are shown in Table 1.

**Table 1:** Final diagnoses of subjects

Diagnosis	Ratio (%)	*n*
Drug-induced parkinsonism	24.1	19
Essential tremor	21.5	17
Dementia without dopaminergic degeneration or mild cognitive impairment	16.5	13
Psychiatric disorders or movement disorder	6.3	5
Normal pressure hydrocephalus	6.3	5
Senile gait or ataxic gait	5.1	4
Dystonia	3.8	3
Vascular parkinsonism without dopaminergic degeneration	2.5	2
Orthostatic tremor	2.5	2
Dizziness	2.5	2
Radiculopathy	1.3	1
Restless legs syndrome or sleep disorder	1.3	1
Cerebellar ataxia	1.3	1
Epilepsy	1.3	1
Wernicke’s encephalopathy	1.3	1
Visual disturbance	1.3	1
Polyneuropathy	1.3	1
Total	100.0	79

### Age and gender effects on DAT binding and cerebral perfusion metabolism

Quantified values of SBRs for dCIT and SUVRs for eCIT are summarized in Table 2. In striatal subregions, SBRs for DAT binding and SUVRs for cerebral perfusion did not show significant gender differences.

**Table 2:** Averaged SBRs and SUVRs in striatal subregions for males and for females

	SBRs for dCIT			SUVRs for eCIT		
	Male (mean ± SD)	Female (mean ± SD)	*P*-value	Male (mean ± SD)	Female (mean ± SD)	*P*-value
Dorsal striatum	4.58 ± 1.10	5.54 ± 1.17	0.560	1.08 ± 0.16	1.20 ± 0.20	0.270
Ventral striatum	4.67 ± 0.98	5.55 ± 1.10	0.508	1.16 ± 0.13	1.24 ± 0.17	0.064
Caudate nucleus	4.29 ± 1.27	5.44 ± 1.30	0.623	0.92 ± 0.19	1.05 ± 0.21	0.540
Putamen	4.72 ± 1.04	5.58 ± 1.12	0.548	1.25 ± 0.14	1.36 ± 0.20	0.119

In SBRs and SUVRs, there was a no significant gender difference. Statistical threshold for pairwise comparison–corrected accounting for Bonferroni (* *P* < 0.05/4)

We also analyzed the multiple regression of quantified SBRs versus age and quantified SUVRs versus age in striatal subregions associated with parkinsonism. The multiple regression analysis revealed coefficients along with their 95% confidence intervals.

**Fig. 2:**
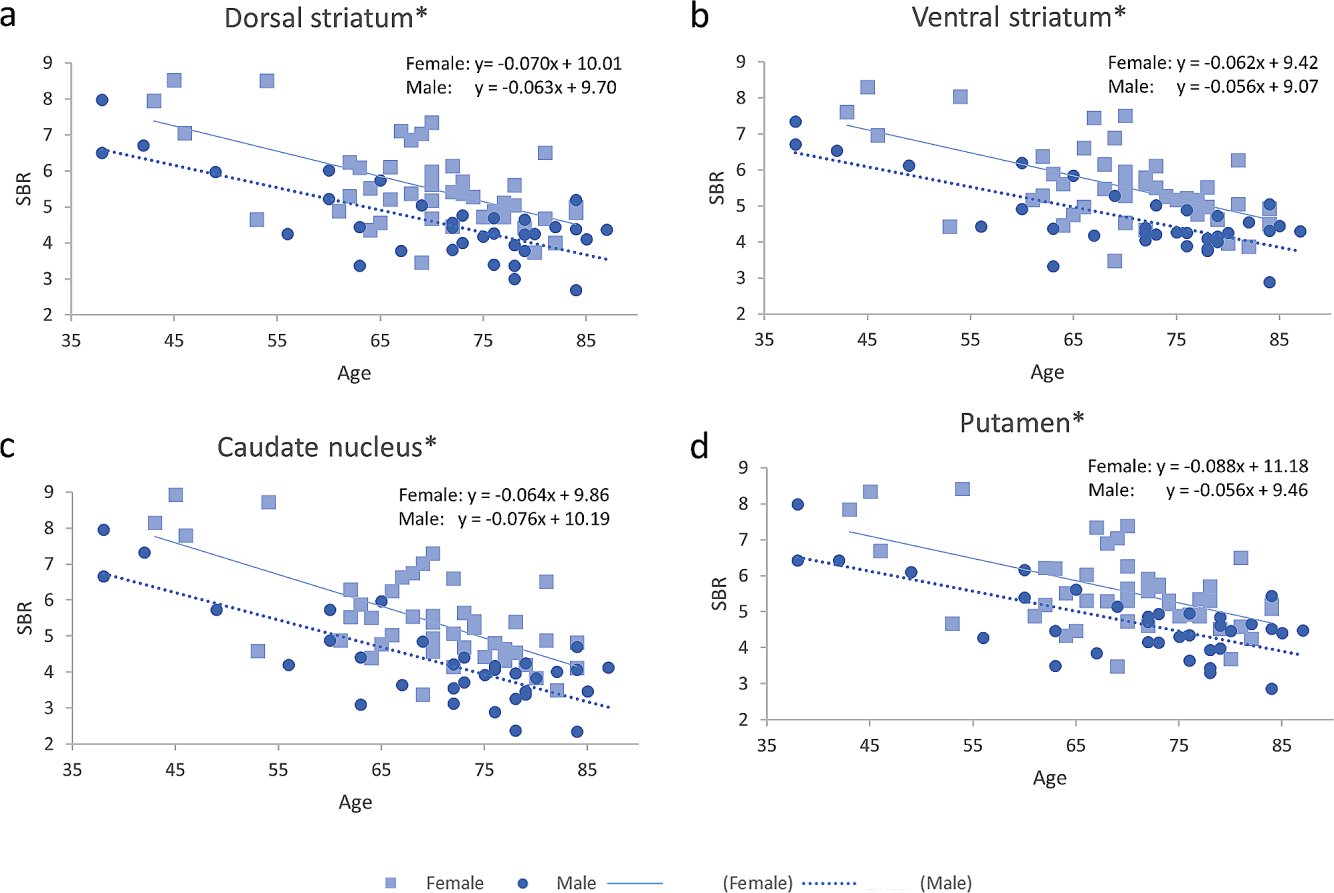
Age and gender effects of SBRs in striatal subregions. The SBRs for dCIT in the (**a**) dorsal striatum, (**b**) ventral striatum, (**c**) caudate nucleus, and (**d**) putamen showed significant negative correlations with age for both genders (female, square and solid line; male, circle and dotted line). The regression lines with slopes and intercepts are displayed. (* *P* < 0.05/4)

In striatal subregions for dCIT, SBRs in the dorsal striatum, ventral striatum, caudate nucleus, and putamen showed significant negative correlation with age for both males and females (Fig. 2). The negative correlation with age were consistent in the dorsal striatal subregions: caudate nucleus (including dorsal anterior, ventral anterior, tail, and body subregions) and putamen (including dorsal anterior, ventral anterior, dorsal posterior, and ventral posterior subregions) (Supplementary Fig. 2).

**Fig. 3:**
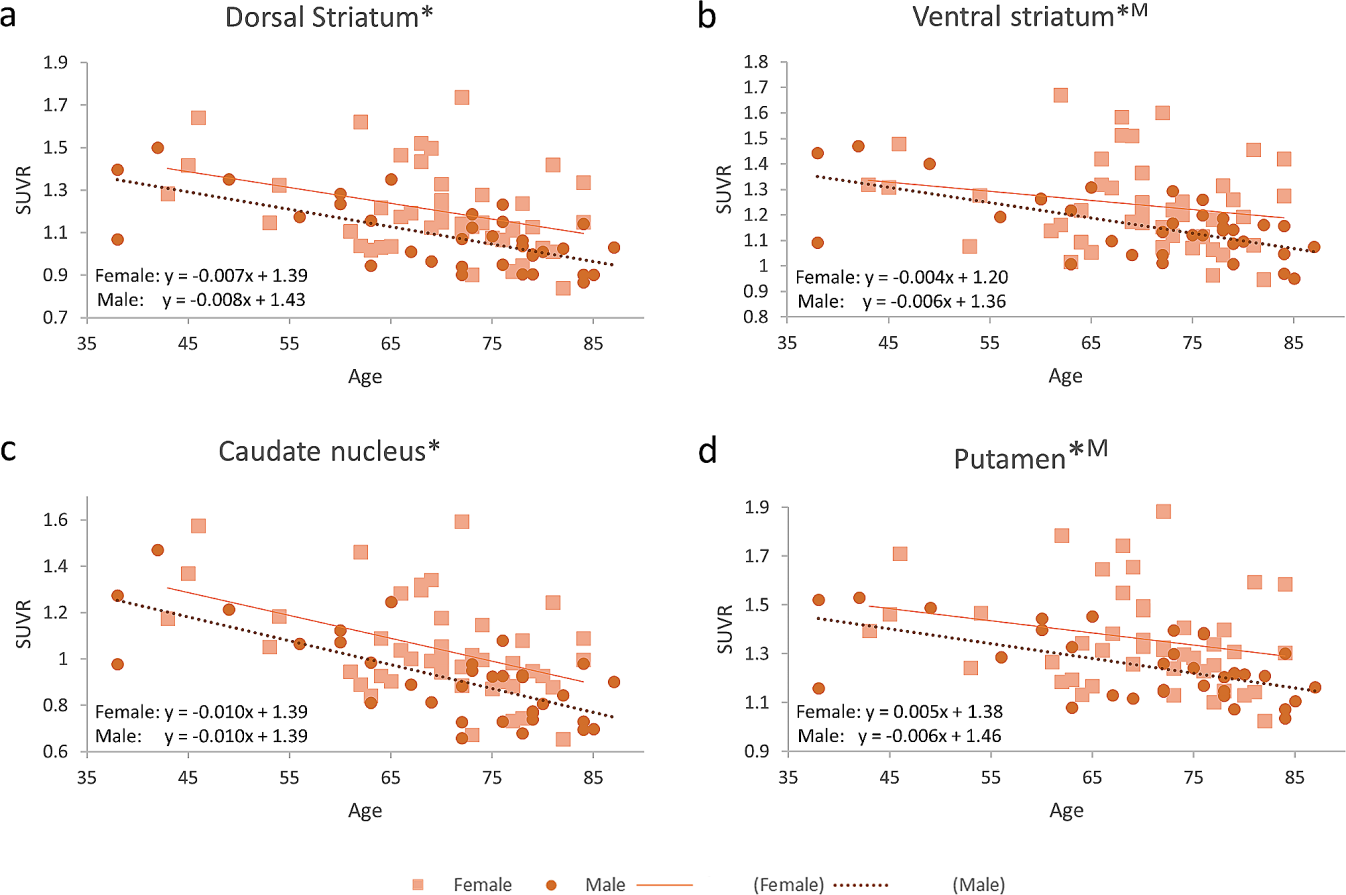
Age effects of SUVRs in striatal subregions. In males, the SUVRs exhibited significant negative correlations with age in the (**a**) dorsal striatum, (**b**) ventral striatum, (**c**) caudate nucleus, and (**d**) putamen, while the SUVRs exhibited significant negative correlations with age in the (**a**) dorsal striatum and (**c**) caudate nucleus for females (female, square and solid line; male, circle and dotted line). The regression lines with slopes and intercepts are displayed. (* *P* < 0.05/4, *M: *P* < 0.05/4 in males only)

In striatal subregions for eCIT, SUVRs in the dorsal striatum, ventral striatum, caudate nucleus, and putamen exhibited a significant negative correlation with age for males, while the SUVRs in dorsal striatum and caudate nucleus exhibited a significant negative correlation with age for females (Fig. 3). The results are summarized in Table 3, depicting the findings from the multiple regression analysis in striatal subregions. Additionally, we examined the extrastriatal perfusion such as the frontal, parietal, temporal, precuneus, and occipital cortices in the multiple regression analysis between SUVRs and age (Supplementary Fig. 3). Only the temporal cortex showed a significant negative correlation between the SUVRs of the eCIT scan and age in both genders.

**Table 3:** Multiple regression analysis in striatal subregions

	SBRs for dCIT								SUVRs for eCIT								
	Male				Female				Male				Female				
	Slope	Inter-cept	*R* ^2^	*P*-value	Slope	Inter-cept	*R* ^2^	*P*-value	Slope	Inter-cept	*R* ^2^	*P*-value	Slope	Inter-cept	*R* ^2^	*P*-value	
Dorsal striatum	-0.063	9.700	0.582	0.000*	-0.070	10.011	0.356	0.000*	-0.008	1.428	0.584	0.000*	-0.007	1.385	0.298	0.008*	
Ventral striatum	-0.056	9.065	0.582	0.000*	-0.062	9.442	0.300	0.000*	-0.006	1.358	0.586	0.000*	-0.004	1.198	0.210	0.154	
Caudate nucleus	-0.076	10.187	0.627	0.000*	-0.064	9.857	0.327	0.000*	-0.010	1.393	0.617	0.000*	-0.010	1.388	0.358	0.001*	
Putamen	-0.056	9.464	0.538	0.000*	-0.088	11.184	0.439	0.000*	-0.006	1.443	0.473	0.000*	-0.005	1.383	0.227	0.077	

Multiple regression analysis (slopes, intercepts, R^2^ values and *P*-values) of SBRs versus age as well as SUVRs versus age for males and females. Statistical threshold for pairwise comparison–corrected accounting for Bonferroni (* *P* < 0.05/4)

### Correlation between DAT binding and cerebral perfusion

**Fig. 4:**
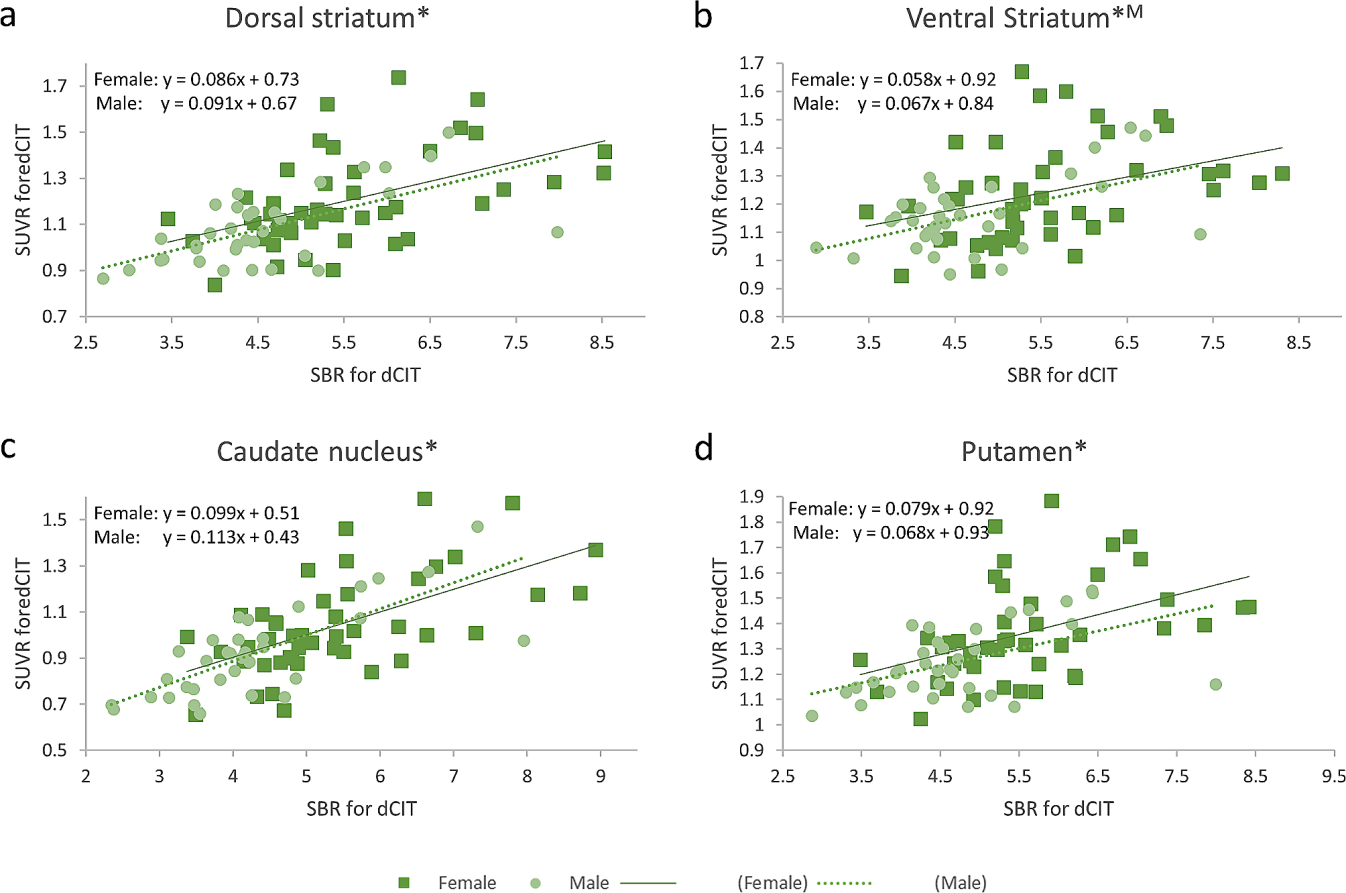
Correlation between SBRs and SVURs in striatal subregions. There were significant positive correlations between SBRs and SUVRs in the (**a**) dorsal striatum, (**b**) ventral striatum, (**c**) caudate nucleus, and (**d**) putamen for males and in the (**a**) dorsal striatum, (**c**) caudate nucleus, and (**d**) putamen for females (female, square and solid line; male, circle and dotted line, * *P* < 0.05/4, *M: *P* < 0.05/4 in males only)

We also analyzed the correlation between SBRs for DAT binding and SUVRs for cerebral perfusion in striatal subregions using quantified data. There were significant positive correlations between SBRs and SUVRs in the dorsal striatum, ventral striatum, caudate nucleus, and putamen for males and in the dorsal striatum, caudate nucleus, and putamen for females (Fig. 4). Pearson correlation parameters for SBRs and SUVRs are summarized in Table 4, along with their 95% confidence intervals.

**Table 4:** Correlation between SBRs and SVURs

	Male		Female	
	Pearson *r*	*P*-value	Pearson *r*	*P*-value
Dorsal striatum	0.626	0.000^*^	0.499	0.000^*^
Ventral striatum	0.520	0.002^*^	0.366	0.013
Caudate nucleus	0.754	0.000^*^	0.599	0.000^*^
Putamen	0.508	0.002^*^	0.445	0.002^*^

Pearson correlation analysis of SBRs and SUVRs in each striatal subregion for males and females. (* *P* < 0.05/4)

## Discussion

In light of the age-related increase in neurodegenerative diseases, the effects of human aging and gender on brain imaging play crucial diagnostic and prognostic roles with advance in imaging technology [21]. In this study, we identified striatal subregion-specific differences according to age and gender in individuals with non-degenerative dopaminergic systems using normal dual-phase ^18^F-FP-CIT scans. We evaluated the effects of age and gender on SBRs for DAT binding and SUVRs for cerebral perfusion through AI-based quantification software. Previous studies reported eCIT within 10 min after intravenous injection well represents cerebral perfusion in the brain [10–12]. Furthermore, recent studies directly comparing eCIT and cerebral perfusion single-photon emission computed tomography (SPECT) revealed the similarities between the two imaging methods [10, 22]. To our knowledge, this was the first study to use dual-phase ^18^F-FP-CIT with a single injection to identify age and gender effects on SBR and SUVR values in striatal subregions using quantitative data.

The results have shown consistent and significant age effects on DAT binding in the dorsal striatum, ventral striatum, caudate nucleus, and putamen for both males and females [23–28]. The striatum consists of the dorsal striatum (including the caudate nucleus and putamen) and the ventral striatum [29]. It mediated the decision-making processes of rewards, behaviors, and emotions, facilitating both reflexive and rational movement and behaviors. The ventral striatum is deeply involved in emotions, reward seeking behavior, and action-outcome learning, while the dorsal striatum is strongly implicated in sensorimotor and movement functions [29, 30]. These findings might serve as evidence explaining the gradual decline in functions such as movement, behavior, cognition, and rewards, associated with aging.

In eCIT scans for cerebral perfusion in striatal subregions, several studies identified shows age and gender effect on cerebral perfusion [27–29]. Previous PET study demonstrated that mean gray matter cerebral perfusion linearly decreased with age especially in the frontal, temporal and parieto-occipital cortices, while white matter cerebral perfusion remained stable with increasing age using oxygen-15 continuous inhalation technique [46]. These observations are consistent with a report of age-related reductions in cerebral perfusion in specific brain regions such as the bilateral cingulate gyri, left inferior gyrus, bilateral medial frontal gyri, left subcallosal gyrus, and left superior temporal gyrus using SPECT [47]. However, recent ^15^O-H_2_O studies suggest that cerebral perfusion may not exhibit an age-related decline after partial-volume correction in healthy individuals [48]. The age-associated decline of cerebral perfusion was detected only at the left superior temporal cortex and no significant difference in mean cerebral perfusion between the elder and younger groups [49]. Previous studies have reported differences in cerebral perfusion between males and females depending on brain subregions [27–29]. In brain SPECT analysis of a total of 128 regions, healthy females showed significantly increased cerebral perfusion in whole brain and 48 concentration regions compared to males, whereas healthy males showed non-significant increases in cerebral perfusion [28]. Additionally, several papers have indicated that cerebral perfusion in the whole brain and limbic region is higher in females compared to males [27–29]. In our study, we found that cerebral perfusion in ventral striatum and putamen decreases more significantly with age in males compared to females, indicating that cerebral perfusion in females is better maintained even as they age.

Some studies suggest that, among women, a fertile life with sufficient female hormones plays an important role in preserving DAT against age-related processes; this protective effect appears to be more pronounced in women than men [25]. Sex hormones have neuroprotective properties against anti-inflammatory anti-apoptotic, and anti-oxidative effects particularly estrogen [31, 32]. Women who experienced early menopause more commonly experienced PD, while those who used estrogens after menopause less frequently developed PD [33]. Early reduction in endogenous estrogen may be associated with an increased risk of developing PD [33].

Loss of DAT binding and decreased cerebral perfusion due to normal aging or pathological degeneration have been reported in some studies, but we have not confirmed which occurs first between loss of DAT binding and decreased cerebral perfusion [24, 31, 34–37]. In this observational study, both males and females showed significant decreases in SBRs for DAT binding according to age. Meanwhile, SUVRs for cerebral perfusion in ventral striatum and putamen displayed a significant decrease only in males. This may suggest that the loss of DAT binding precedes the decrease in cerebral perfusion, but further longitudinal research is required.

Although the causal relationship between DAT binding loss and perfusion decline remains unclear, we founded positive correlations between SBR for DAT binding and SUVRs for cerebral perfusion in each striatal subregion: dorsal striatum, ventral striatum, caudate nucleus, and putamen for males and dorsal striatum, caudate nucleus, and putamen for females. Although the ventral striatum did not show statistical significance in female, it exhibits a marginal *p*-value of 0.013. This indicated a coupled change in DAT binding and cerebral perfusion during the normal aging process rather than as a result of pathological degeneration.

In this study, quantitative data were obtained through AI-based commercial software rather than traditional measurement methods, such as Statistical Parametric Mapping or FreeSurfer software, saving time and effort. Additionally, given the high agreement between values extracted by the existing gold-standard method and AI-based extraction values reported [38], we did not recheck the agreement between values obtained by the traditional method and the quantified values in this study.

There were several limitations to the study. It was assumed that all participants had non-degenerative parkinsonism. All participants were excluded based on the final diagnosis of parkinsonism with dopaminergic system degeneration, but we did not conduct extensive neuropsychological testing for various cognitive impairments. Thus, we cannot fully exclude all kinds of cognitive disorders in this cohort. There were several limitations to the study. It was assumed that all participants had non-degenerative parkinsonism. All participants were excluded based on the final diagnosis of parkinsonism with dopaminergic system degeneration, but we did not conduct extensive neuropsychological testing for various cognitive impairments. Thus, we cannot fully exclude all kinds of cognitive disorders in this cohort. However, this study only included patients who were followed up for at least 2 years after onset, with some observed for several years or more. It is remarkable that 16 patients with normal dCIT and eCIT scans were ultimately diagnosed with neurodegenerative parkinsonism and were excluded from the analysis. Among these excluded patients, follow-up results revealed final diagnoses not only of PD but also multiple system atrophy, progressive supranuclear palsy, corticobasal degeneration, and dementia with Lewy bodies. Moreover, assuming eCIT images reflect cerebral perfusion, we performed eCIT imaging exclusively without comparing it to perfusion images. However, many studies have already reported similarities between eCIT and cerebral perfusion, suggesting that a separate analysis may not be necessary [10, 13]. Another limitation is that we used different striatal atlases for Melbourne in dCIT and for AAL3 in eCIT. It is well known that the anterior-posterior gradient in the putamen is the most noticeable feature associated with disease progression in PD [39, 40]. Therefore, when analyzing the SBR of DAT binding in dCIT, we used the Melbourne atlas to demonstrate the effect of ageing on DAT binding in the striatal subregions in more detail. We considered that there were no significant differences between the two atlases in striatum.

## Conclusions

We found that the patterns observed regarding age and gender effects on DAT binding and cerebral perfusion have a significant impact on the brain. For SBRs of DAT binding in striatal subregions, we demonstrated a negative acceleration of the aging effect in the dorsal striatum, ventral striatum, caudate nucleus, and putamen for both males and females. For SUVRs of cerebral perfusion in striatal subregions, we observed negative accelerations of the aging effect in the dorsal striatum, ventral striatum, caudate nucleus, and putamen for males as well as in the dorsal striatum and caudate nucleus for females. The study revealed that the SBR and SUVR are positively correlated in the dorsal striatum, ventral striatum, caudate nucleus, and putamen for males and in the dorsal striatum, caudate nucleus, and putamen for females. These observations indicate that DAT binding and cerebral perfusion should be considered concerning age and gender effects. The quantified data from dual-phase ^18^F-FP-CIT can be beneficial for determining normal and abnormal conditions in image interpretation in clinical settings, by considering variations associated with age and gender.

### Electronic supplementary material

Below is the link to the electronic supplementary material.

Supplementary Material 1

Supplementary Material 2

Supplementary Material 3

## Data Availability

Data supporting this study will be made available upon reasonable request.
