# Supplementary figures and images for: Age and gender effects on striatal dopamine transporter density and cerebral perfusion in individuals with non-degenerative parkinsonism: a dual-phase 18F-FP-CIT PET study
Source: EJNMMI Res. 2024 Jul 17;14:65. doi: 10.1186/s13550-024-01126-1 (PMC11254898; doi:10.1186/s13550-024-01126-1)

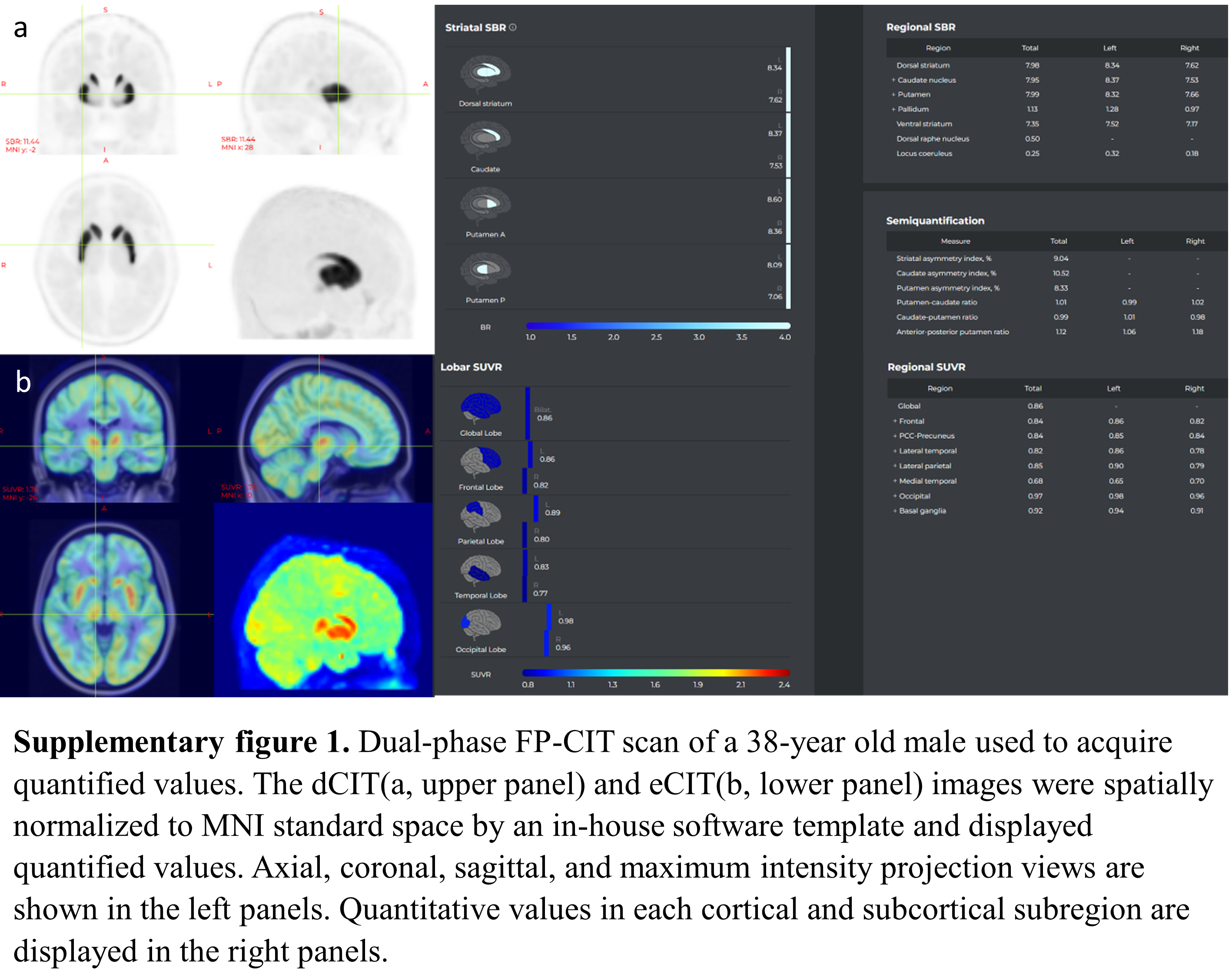

Supplement: Supplementary file 1 — Supplementary Material 1 [file 13550_2024_1126_MOESM1_ESM.tif]

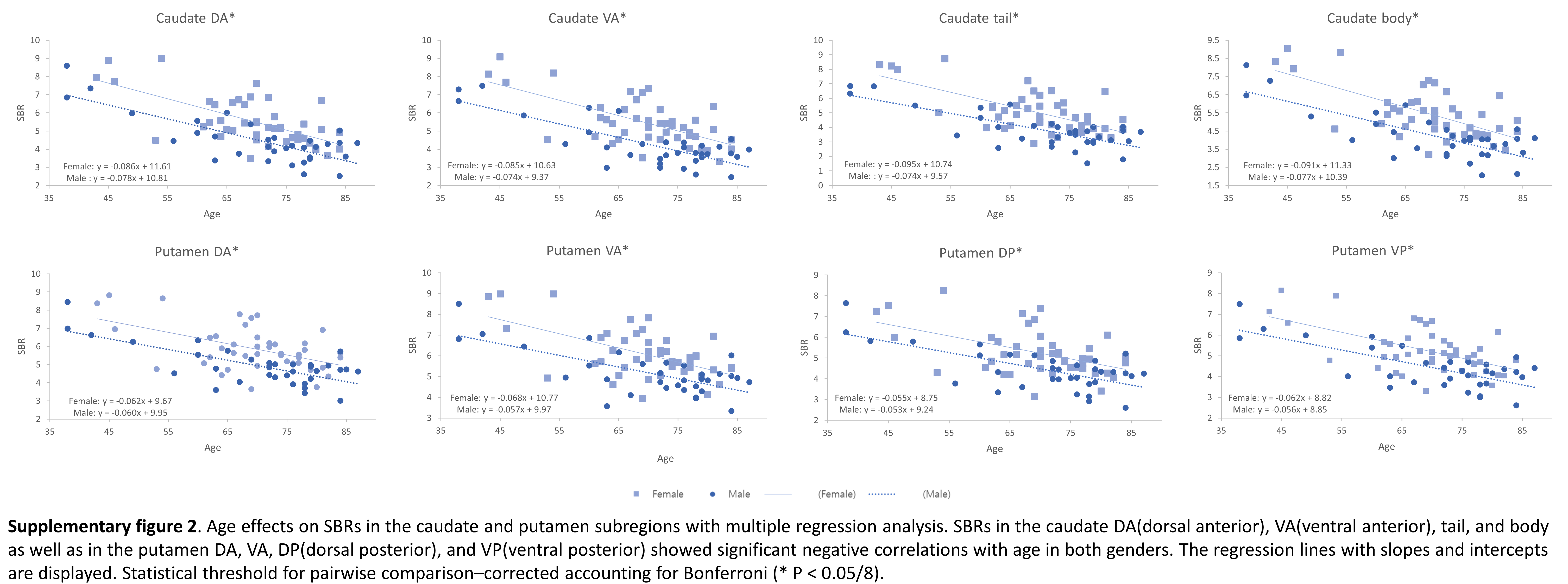

Supplement: Supplementary file 2 — Supplementary Material 2 [file 13550_2024_1126_MOESM2_ESM.tif]

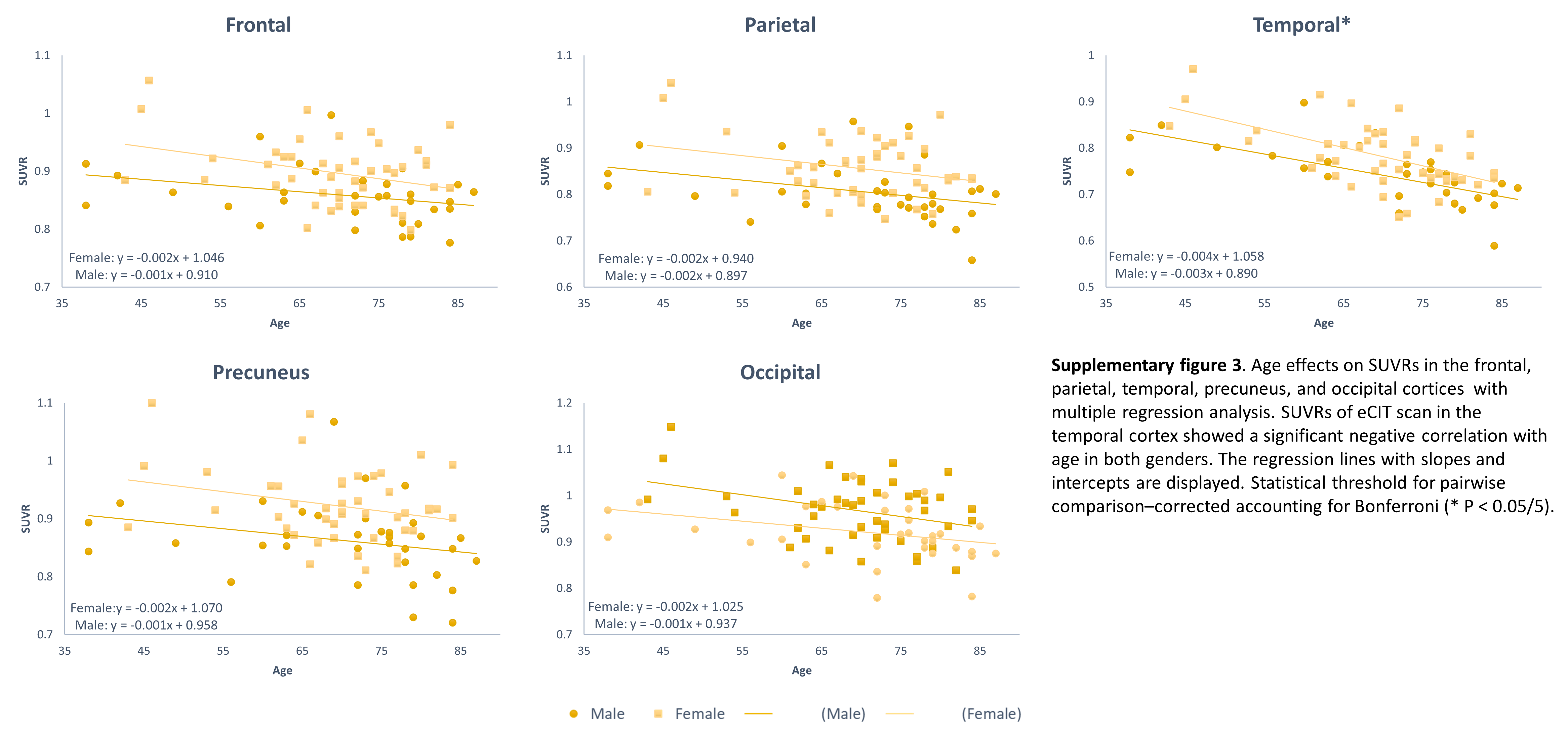

Supplement: Supplementary file 3 — Supplementary Material 3 [file 13550_2024_1126_MOESM3_ESM.tif]
